# Enablers and barriers to physical activity among older adults of low socio-economic status: a systematic review of qualitative literature

**DOI:** 10.1186/s12966-025-01753-4

**Published:** 2025-06-23

**Authors:** Olivia S. Malkowski, Jessica Harvey, Nick P. Townsend, Mark J. Kelson, Charlie E. M. Foster, Max J. Western

**Affiliations:** 1 Centre for Motivation and Behaviour Change, Department for Health, University of Bath, Bath, UK; 2 Centre for Exercise, Nutrition and Health Sciences, School for Policy Studies, University of Bristol, Bristol, UK; 3 Institute of Data Science and Artificial Intelligence, Department of Mathematics, University of Exeter, Exeter, UK

**Keywords:** Older adults, Physical activity, Systematic review, Qualitative research, Health inequalities, Socio-economic status

## Abstract

**Background:**

Understanding the factors influencing physical activity in older adults, and whether they vary according to socio-economic status (SES), could help to inform interventions that are effective in reducing inequalities and improving the quality of life of an ageing population. This systematic review aimed to synthesise the qualitative evidence on the modifiable enablers of, and barriers to, physical activity based on low-SES older adults’ perspectives in the United Kingdom (UK). A secondary aim was to identify and summarise differences in physical activity enablers and barriers between older adults of low and high SES.

**Methods:**

We searched five electronic databases from inception to December 2023 for studies conducted among UK-based, community-dwelling older adults aged 60+ years including qualitative methods, with results reported by SES. We excluded hospitalised or institutionalised participants. Risk of bias was assessed with the Mixed Methods Appraisal Tool, and framework synthesis was applied using the Capability, Opportunity, Motivation, and Behaviour (COM-B) model.

**Results:**

Thirty studies were included in the review, of which five specifically examined inequalities in physical activity enablers and barriers. Low-SES older adults’ physical capability was influenced by fitness, mobility, and general health, while their psychological capability was shaped by knowledge and behavioural regulation. Physical opportunity was characterised by safety, pedestrian infrastructure, access to physical activity opportunities and daily destinations, environmental quality, and aesthetics. Themes under social opportunity encompassed social support, social norms, social engagement, and dog ownership. Physical activity was motivated by reflective processes, such as outcome expectancies, self-efficacy, and attitudes, as well as automatic processes, including habits, lack of time, and enjoyment. Few studies investigated differences between participants of low and high SES, with those that did predominantly pointing to disparities in the physical or built environment.

**Conclusions:**

This meta-synthesis of qualitative literature identified a wide range of interacting factors influencing physical activity across socio-ecological and COM-B domains, underscoring whole-system interventions as a potential approach to stimulate meaningful and sustained change. Future research could report results by SES to enhance our understanding of inequalities and ensure that low-SES older adults are represented in the development and evaluation of interventions targeting improvements in physical activity.

**Supplementary Information:**

The online version contains supplementary material available at 10.1186/s12966-025-01753-4.

## Background

The health benefits of physical activity are well documented, with decreased risk of numerous chronic diseases and premature mortality attributed to those people who exercise and move regularly relative to those who lead more sedentary lives [1]. For older adults aged 60 years and above, the benefits of maintaining physical activity are particularly pronounced as the body attempts to combat declines in physical function that accelerate in later life [2]. However, population-based data suggest that older adults are the least active segment of society, with only 19% of 65–74-year-olds, and 10% of those aged 75 years and above, meeting governmental guidelines for physical activity in the United Kingdom (UK) [3]. Moreover, a socio-economic gradient in physical activity exists across the lifespan, which increases up to the age of 85 years, with individuals on high income meeting recommendations at a greater rate than those on low income [4, 5]. Similar patterns exist for other indicators of socio-economic status (SES), including education and occupational class [6–8]. Understanding the factors that influence physical activity behaviour in older adults, and whether they differ according to SES, is paramount to developing policies and interventions that are effective in reducing inequalities and improving the health and quality of life of an ageing population.

A common way to categorise the influences on individuals’ physical activity uses the socio-ecological model of health, a multilevel framework that illustrates the complex interplay between the intrapersonal, interpersonal, environmental, and public policy factors shaping people’s behaviour [9]. Specific influences that fall within these categories that support engagement in the behaviour are frequently called enablers, whereas factors that impede engagement in the behaviour are often referred to as barriers. A contemporary framework to classify socio-ecological enablers and barriers is the Capability, Opportunity, Motivation, and Behaviour (COM-B) model [10]. The COM-B model posits that three conditions are essential for any behaviour, such as physical activity, to change: notably, physical (e.g., skills and mobility) and psychological (e.g., knowledge and memory) *capability*, physical (e.g., environmental context and resources) and social (e.g., cultural norms and support) *opportunity*, as well as reflective (e.g., beliefs about consequences) and automatic (e.g., enjoyment and habits) *motivation*. According to the model, these components interact, such that a more pleasant environment for walking (physical opportunity) may enhance one's intention to walk (reflective motivation). Therefore, to promote physical activity, one or more of these components are likely to need to be modified [11].

While several systematic reviews have explored factors associated with older adults’ capability, opportunity, and motivation to engage in physical activity behaviour [12–15], little work has been done to understand if these factors differ by SES. This is important as policies and interventions born out of an evidence base largely skewed towards more affluent populations risk widening rather than narrowing inequalities in health outcomes. A pertinent example of this relates to digital physical activity interventions, which across all age groups, appear to be effective in higher but not lower-SES groups [16]. To address this gap in the literature, we recently conducted a systematic review with meta-analysis exploring the correlates of physical activity behaviour among UK older adults of low versus high SES [17]. After extracting quantitative data from 77 studies, pertinent influences on physical activity behaviour for low-SES older adults included physical function, social participation, and perceived general health, though many of these were broadly equivalent for participants of higher SES. The results suggest that it may not be the mechanisms underpinning physical activity that drive inequalities in behaviour, but rather an imbalance in the prevalence of these correlates (e.g., a greater proportion of higher-SES older adults reporting good social participation or perceived general health relative to their low-SES counterparts).

There are, however, several shortcomings of solely using quantitative data to understand the complex nature of physical activity behaviour, and how its antecedents might differ between people of low and high SES, that warrant consideration when interpreting these review findings. Firstly, a large proportion of the evidence used in the systematic review with meta-analysis was derived from cohort studies on the dynamics of people’s health, social, and economic circumstances, preventing an explicit focus on physical activity behaviour. Reviews of the older adult population have identified a host of physical activity correlates [18], yet many factors, particularly intrapersonal ones akin to motivation and attitudes were seldom captured among datasets where stratified analyses by SES were or could be conducted. Secondly, the meta-analyses were inevitably limited by measures used in the original quantitative studies. Notably, environmental exposures such as green space or physical activity facilities were measured in terms of access, volume, and proximity, rather than quality, despite the latter likely playing an important role. Without gaining insight into how these quantitative correlates are perceived by older adults, our understanding will be restricted to ‘what’ they are and not ‘why’ they manifest as inequalities in behavioural outcomes. To overcome such challenges and gather information on older adults’ subjective experiences of physical activity, one might therefore use a qualitative meta-synthesis.

The current review aimed to systematically examine and synthesise qualitative evidence on the modifiable enablers of, and barriers to, physical activity based on low-SES older adults’ perspectives. A modifiable factor was defined as any characteristic susceptible to change through policies, the physical and social environment, and/or an individual’s own choices and efforts. Further, this paper aimed to identify and summarise differences in physical activity enablers and barriers between older adults of low and high SES. We focus on the UK, both for pragmatic reasons, and because each country is encouraged to identify and implement policy actions tailored to their national context to increase overall levels of physical activity and decrease rates of physical inactivity [19].

## Methods

### Search strategy

The present article is reported in accordance with the Preferred Reporting Items for Systematic Reviews and Meta-Analyses (PRISMA) statement (Additional file 1) [20]. The protocol, which details the overlap between this study and the concurrent systematic review of quantitative evidence, was registered on the International prospective register of systematic reviews (PROSPERO; CRD42022351708) [21]. Specifically, while we sought to identify studies exploring the correlates, determinants, enablers, and/or barriers underlying older adults’ physical activity participation in a singular search strategy, we decided *a priori* that studies presenting statistical associations between modifiable correlates or determinants and physical activity behaviour would be synthesised and published separately. The search was carried out by OSM in five electronic databases (18 December 2023; see Additional file 2 for the full search strategy): MEDLINE (PubMed interface), Embase (Embase.com interface), Web of Science, Cochrane Central Register of Controlled Trials (CENTRAL), and Scopus. No restriction was placed on dates of coverage. Trial registries (International Standard Randomised Controlled Trial Number [ISRCTN] registry; ClinicalTrials.gov) and the Open Science Framework were searched by OSM for grey literature. Existing reviews, and the reference lists of included studies, were manually screened by OSM to identify other relevant articles.

### Selection criteria

This review was restricted to original, peer-reviewed studies. Inclusion criteria were: (1) studies involving community-dwelling UK older adults aged 60+ years, including those that examined the UK and/or older adult population as a sub-group where extrapolation was possible (i.e., studies could recruit and involve individuals aged below and above 60 years as long as results were reported separately for older adults aged 60+ years, in which case only data relating to those participants who met the eligibility criteria were considered in the review); (2) studies on one socio-economic sub-group (i.e., all low-SES participants, or all high-SES participants) or with results reported by SES; and (3) qualitative studies or mixed methods studies with a qualitative component. For mixed populations (i.e., studies where only some participants met the inclusion criteria) where results were not disaggregated by age, the study was included if the mean age was 60 years or above and no participants were aged less than 50 years. Exclusion criteria were: (1) studies focusing exclusively on hospitalised or institutionalised participants; and (2) studies published in a language other than English. Conference abstracts, dissertations and theses, editorials, opinions, letters, trial protocols, reviews, and case reports were also excluded.

### Data screening and extraction

The search results were imported into EndNote reference management software [22] and Covidence systematic review software [23], where duplicates were automatically removed. We checked for additional duplicate records using EndNote, following the method outlined by Bramer and colleagues [24], and manually marked newly identified duplicates within Covidence. All the retrieved articles were independently screened for eligibility by OSM, in two stages: (1) titles and abstracts; and (2) full texts. At each stage, 30% of articles were double screened (there was over 90% proportionate agreement at each stage) by two other review authors (MJW and JH; 15% each based on the original search results), working independently. Disagreements at the title and abstract stage were resolved via discussion between the two reviewers concerned, whereas conflicts at the full text stage were resolved via discussion with a fourth review author (NPT). Any decisions made during this process were applied uniformly by OSM to the remaining articles. Data extraction was conducted in Covidence by OSM (100%) using a pre-determined template (Additional file 3); two review authors (MJW and JH) independently controlled and validated a 15% random sample of completed data extraction templates each (i.e., reviewed, but did not independently extract data using the pre-determined template). At this stage, the types of factors, and how these factors influenced participants’ physical activity behaviour, were extracted as defined by the authors of the original studies.

### Risk of bias assessment

To account for the range of study designs identified and included in the systematic review (when considered in tandem with the quantitative evidence, which is reported separately), the Mixed Methods Appraisal Tool (MMAT) was used to assess risk of bias [25]. OSM appraised each study independently. Two other review authors (MJW and JH) each independently rated a 15% random sample of included studies (there was over 80% proportionate agreement). The results of these dual appraisals were compared, and conflict resolution was undertaken via discussion with NPT; single-screened papers were subsequently verified by OSM to ensure consistency in approach. The MMAT is rated according to the following criteria for qualitative studies: appropriateness of the qualitative approach; adequacy of data collection and analysis methods; substantiation of the interpretation of the results by data; and coherence between qualitative data sources, collection, analysis, and interpretation. For mixed methods studies, the criteria comprise: the provision of an adequate rationale for using a mixed methods design; effective integration of the different components; adequate interpretation of the outputs of the integration; suitable explanations of divergences and inconsistencies between quantitative and qualitative results; and adherence of the different components to the quality criteria of each tradition.

The response options are “yes”, meaning the criterion is met, “no”, meaning the criterion is not met, or “can’t tell”, meaning there is not enough information to judge if the criterion is met or not. The MMAT does not compute a summative numerical score; therefore, a more detailed presentation of the ratings for each criterion is provided. To ensure ratings could be applied uniformly across reviewers, modified indicators and concrete thresholds were developed by OSM for any ambiguous criteria (Additional file 4).

### Data analysis

Data analysis was conducted jointly by OSM and MJW. The results sections of the reviewed studies were assessed by means of framework synthesis [26]. Firstly, extracted data were read thoroughly and deductively coded using the COM-B model as a means of organising the data for subsequent interpretation. Themes and sub-themes were developed based on the six sub-components of the COM-B model, including physical capability, psychological capability, physical opportunity, social opportunity, reflective motivation, and automatic motivation [10]. Extracted data were then re-visited line by line by OSM to ensure a good fit. An element of flexibility was maintained to account for any new or unexpected themes. The results were presented narratively, and the findings illustrated using participant quotes. We also compared findings from older adults of low versus high SES across the themes and sub-themes named in this initial stage, for the studies that specifically aimed to examine inequalities in physical activity enablers and barriers.

## Results

### General characteristics

**Fig. 1:**
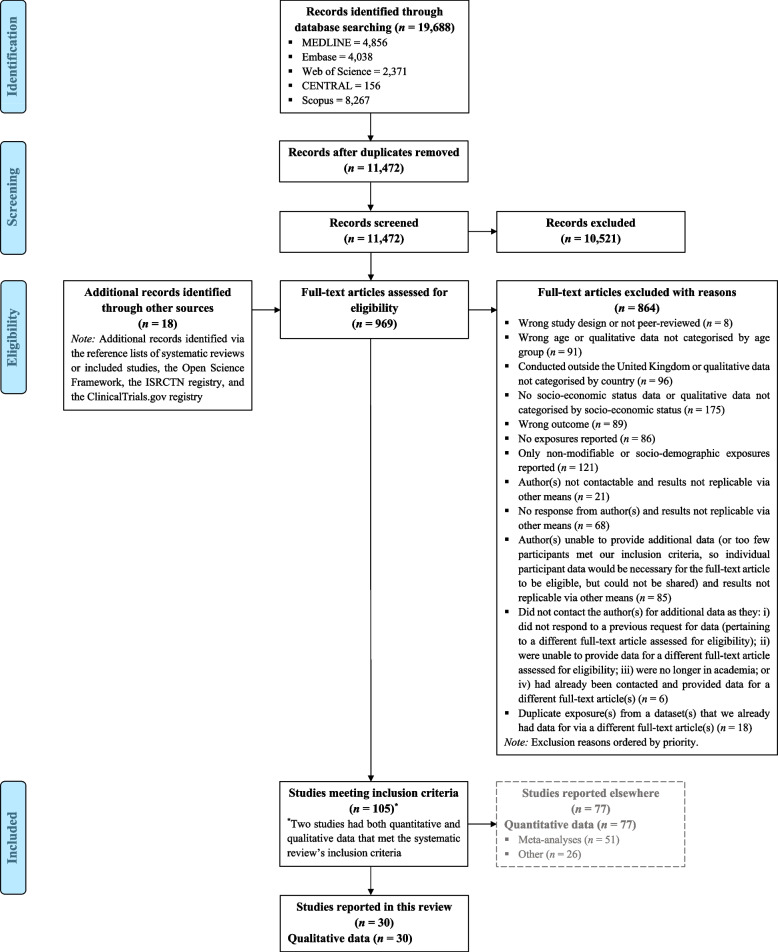
PRISMA flow diagram of the study selection process. *Note:* CENTRAL, Cochrane Central Register of Controlled Trials; ISRCTN, International Standard Randomised Controlled Trial Number; PRISMA, Preferred Reporting Items for Systematic Reviews and Meta-Analyses

We screened 11,472 records at the title and abstract stage and 969 records at the full text stage. Thirty studies were included in this synthesis, published between 1996 and 2023 [27–56]. A complete flow diagram of the study selection process is displayed in Fig. 1. The overall sample sizes ranged from 7 to 75 participants across all age ranges included in the studies, and from at least 1 to 52 participants when limited to those aged 60+ years meeting our inclusion criteria (Additional file 5). It is important to note that many studies reported findings across multiple components and/or sub-components of the COM-B model. As such, the total number of participants differs for each theme and/or sub-theme identified. Most studies (*N* = 28) recruited male and female participants [27–49, 51, 53–56], while two focused only on females [50, 52]. Fifteen studies recruited individuals of low and high SES [27–33, 36, 37, 40, 44, 46, 50, 53, 56] and the other fifteen recruited solely those of low SES [34, 35, 38, 39, 41–43, 45, 47–49, 51, 52, 54, 55]. When confined to participants aged 60+ years meeting our inclusion criteria, twelve studies contained data on older adults of low and high SES [27–33, 36, 37, 44, 46, 50]. Among these, five aimed to compare enablers of, and barriers to, physical activity in participants of low versus high SES [27–31]. Of the remaining studies, sixteen included data (e.g., quotes) on only low-SES participants [34, 35, 38, 39, 41–43, 45, 47–49, 51–55], and two only on high-SES participants (please note that, while these two studies were eligible based on our inclusion criteria, results are not reported in the text, as we focus only on high-SES older adults if a comparison can be drawn with low-SES older adults) [40, 56].

### Methodological characteristics and risk of bias assessment

**Fig. 2:**
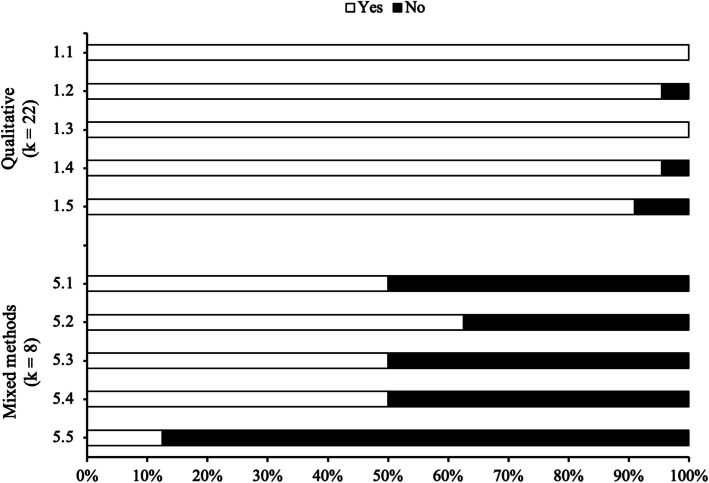
Risk of bias of the included studies. *Note:* Risk of bias was assessed using the Mixed Methods Appraisal Tool, Version 2018. k, number of studies. “Yes” rating: the criterion is met; “No” rating: the criterion is not met. Criterion 1.1: Is the qualitative approach appropriate to answer the research question? Criterion 1.2: Are the qualitative data collection methods adequate to address the research question? Criterion 1.3: Are the findings adequately derived from the data? Criterion 1.4: Is the interpretation of results sufficiently substantiated by data? Criterion 1.5: Is there coherence between qualitative data sources, collection, analysis and interpretation? Criterion 5.1: Is there an adequate rationale for using a mixed methods design to address the research question? Criterion 5.2: Are the different components of the study effectively integrated to answer the research question? Criterion 5.3: Are the outputs of the integration of qualitative and quantitative components adequately interpreted? Criterion 5.4: Are divergences and inconsistencies between quantitative and qualitative results adequately addressed? Criterion 5.5: Do the different components of the study adhere to the quality criteria of each tradition of the methods involved?

From the thirty included studies [27–56], twenty-two exclusively used qualitative methods [27, 28, 31–33, 35–39, 41, 42, 44–46, 48, 50, 51, 53–56], whereas eight combined qualitative and quantitative methods (mixed methods studies) [29, 30, 34, 40, 43, 47, 49, 52]. The qualitative data included in the review were predominantly collected via interviews (*N* = 19) [27, 30–34, 37–40, 43, 46, 49–51, 53–56] and focus groups (*N* = 6) [28, 35, 41, 42, 48, 52], although some studies used a combination of interviews, focus groups, observations, workshops, and/or questionnaires with qualitative sections (*N* = 5) [29, 36, 44, 45, 47]. In eight studies [34, 43, 45, 47, 49, 52, 55, 56], the qualitative procedures were nested within randomised trials or natural experiment studies (e.g., process evaluations). Using the MMAT (Fig. 2), the most common methodological limitation among qualitative studies was a lack of coherence between data sources, collection, analysis, and interpretation (criterion 1.5), whereas for mixed methods studies, the weakest criterion (5.5) was having the qualitative and/or quantitative components rated of low quality.

### Enablers and barriers among older adults of low SES

**Fig. 3:**
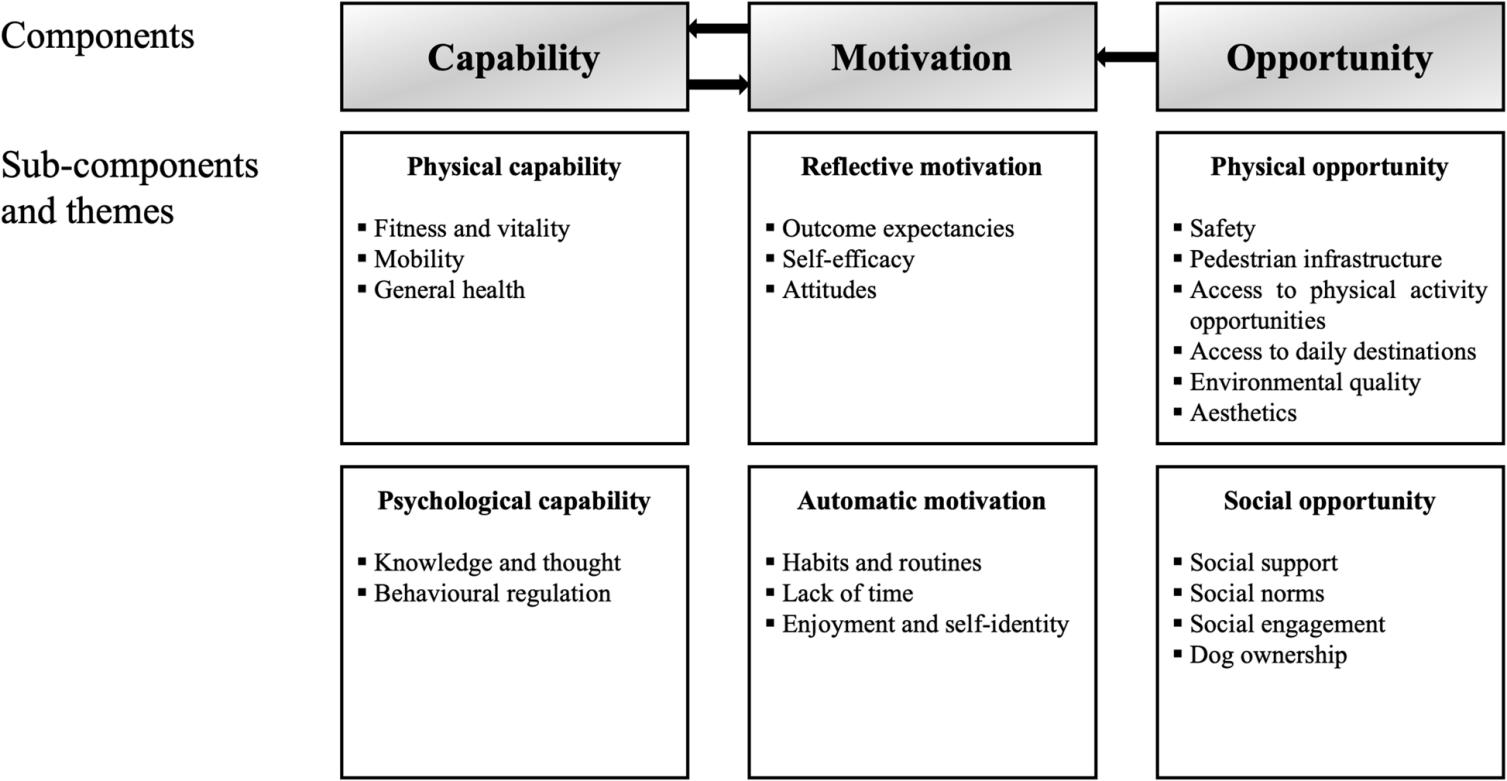
Enablers and barriers to physical activity among low-SES older adults mapped onto the COM-B model [10]. *Note:* SES, socio-economic status; COM-B, Capability, Opportunity, Motivation, and Behaviour

The themes are mapped against the COM-B model and summarised in Fig. 3. Most reviewed studies had findings classified within physical opportunity (*N* = 21) and social opportunity (*N* = 21), followed by reflective motivation (*N* = 19), physical capability (*N* = 15), automatic motivation (*N* = 15), and psychological capability (*N* = 8).

#### Capability

Themes relevant to the capability component of the COM-B model are displayed in Table 1, along with select participant quotes.

**Table 1:** Factors influencing low-SES older adults’ capability to engage in physical activity

**COM-B sub-component**	**Themes**	**Extracts and/or quotes**	**Contributing studies**
***Physical capability***	Fitness and vitality	“A few men were also convinced that they did not need to do regular exercise as they were *'fit enough to do exercises'*” [32] *“Because it was a very physically and mentally demanding job I didn't really have the time or the energy… I do believe I’m more energised since I finished work because it was very challenging mentally and physically and I don’t have that anymore so I can, you know, I can divert all those energies elsewhere”* [33]	[32–36, 38]
	Mobility	*“…as the body shuts down, I don’t mean that too literally, you know, so you don’t play the tennis but you can still play bowls and you still can do your walking and I can still play with the grandchildren so physically I can do all those things still…”* [36] “Margaret is in her seventies and suffers from poor mobility but lives alone in Swffryd. Although she has a wheelchair she often cannot use it and has to walk as she feels that buildings and public transport often do not cater for disabled people (e.g., steps and no ramp). Margaret feels that she is unable to do any physical activity because of these issues.” [39]	[34, 36, 38, 39, 41–46]
	General health	*“Although I haven’t done it [physical activity] in about 2 months. Erm I think that one day I just done a bit too much and then fluid gathered in my knees. So, I need to be careful not to over do it.”* [47] *“A whole lot of them are diabetic... [member of the healthy living center] is diabetic. [Member of the healthy living center] because she is not fit she doesn’t exercise, she would love to exercise. It’s her health that keeps her from doing it”* [28]	[28, 36, 39, 43, 44, 46, 47]
***Psychological capability***	Knowledge and thought	*“... but I don’t know what 300 steps would mean. Would that mean it’s good or bad?”* [47] *“You’ve just given me thought there to think about actually, I haven’t really given that a thought to be honest… it’s rather big question that to be quite honest, stirred my imagination a bit”* [33]	[28, 33, 41, 43, 47–49]
	Behavioural regulation	*“I know my own diet and I know what to do… I know when to eat, what to eat, how to eat, go for a walk round.”* [49]	[49, 50]

*SES* socio-economic status, *COM-B* Capability, Opportunity, Motivation, and Behaviour

##### Physical capability

Themes pertaining to the physical capability sub-component included: (1) fitness and vitality; (2) mobility; and (3) general health. A few individuals who evaluated their own physical fitness as sufficient believed they did not need to exercise, whereas working older adults mentioned job-related fatigue and lack of vitality as barriers to physical activity. Poor mobility altered older adults’ expectations of their capabilities. Some participants discussed replacing moderate-to-vigorous sports with light intensity alternatives as a solution. Many relied on family to go out (see also social support), as the environment did not cater for people with functional limitations. In addition, older adults’ general health influenced their willingness and ability to be active. Participants with existing conditions experienced symptoms which restrained the frequency, intensity, duration, and mode of physical activity they could do and suppressed their motivation. For others, deteriorating health did not preclude them from getting on with their lives.

##### Psychological capability

Themes grouped under psychological capability were: (1) knowledge and thought; and (2) behavioural regulation. Several older adults lacked awareness of the physical activity guidelines, though a growing realisation of their physical boundaries provided a foundation for instigating positive behaviour changes. Giving conscious thought to physical activity strategies ahead of retirement reinforced participants’ knowledge. In a few studies, older adults cited behavioural regulation, namely their ability to manage aspects of their lifestyle, as a motive for physical activity uptake and maintenance.

#### Opportunity

Due to the high number of themes and sub-themes identified, the review findings for the opportunity component of the COM-B model are reported separately for the physical opportunity and social opportunity sub-components in the following section.

##### Physical opportunity

Table 2 presents the themes, sub-themes, and quotes related to physical opportunity. The six themes organised under this sub-heading were: (1) safety; (2) pedestrian infrastructure; (3) access to physical activity opportunities; (4) access to daily destinations; (5) environmental quality; and (6) aesthetics.

**Table 2:** Factors influencing low-SES older adults’ physical opportunity to engage in physical activity

**Themes**	**Sub-themes**	**Extracts and/or quotes**	**Contributing studies**
***Safety***	Traffic-related safety	*“Erm [coughs] I think that is becoming increasingly difficult as the roads, the roads have got so busy now. Aye. It’s not so pleasant.”* [47] *“…I think the roads are dangerous…I don’t drive, but being a passenger, it’s bad enough…they’re not made, are they, for the volume of traffic? You’re all right on the cycle lane, you know if you have a cycle lane, but it’s when you come to lights or a roundabout…”* [42]	[27–31, 42, 43, 45, 47, 51–53]
	Crime-related safety	*“They stand about in gangs too. I definitely wouldn’t feel safe going out at night especially if there are drugs about. No way”* [28] *“…there’s more security’cause … people can see what’s going on.”* [52]	
***Pedestrian infrastructure***	Quality and maintenance	*“when we walk up here, we have to be very careful! Broken and broken (pavements in Alum Rock Road)! This is disgusting!”* [29] *“Even some of the footpaths when walking, you go up and down like a yo-yo on them, you would break your neck very easily on them, they are not very safe you know. They would need to be new tarmacked. Round where we live”* [28]	[27–29, 45, 47, 53]
	Slopes and curbs	*“they moved bus stops and it … [u]sed to stop outside the bookies but now they've taken it up to the cash and carry so it's quite a wee walk back for me and it's a bad pavement and a bad road and a big kerb, so that's awkward for me.”* [45] *“Broken pavements and slope! It would definitely mean you have to watch!”* [29]	
	Road crossings	*“Eh, no. Never go in them […] I'll take a long way round rather than go in an underpass […]”* [53]	
***Access to physical activity opportunities***	Access to recreational facilities	*“We don’t have anything local, nowhere to go to, we only have this wee centre here and there’s only a certain amount of things you can do”* [28] *“Oh, Tuesdays used to be enormous. That's the biggest loss in this village. We had a tennis court. We had a bowling green. We had two football teams and we weren't any down [short of players].”* [35]	[27–31, 34, 35, 38, 39, 41–43, 46–48, 51–54]
	Walking and cycling infrastructure	*“You can get from A to Z (in my neighborhood), you can go many ways. You can walk many ways!”* [30] “To an outsider visiting the area the Loop Line appears to offer excellent facilities for cycling and walking in peaceful, traffic-free surroundings. However, for local people, it was a different story: *'…you’re talking about cycle paths, but you couldn’t go there on your own…it’s not very nice, there’s horrible people on the cycle paths'*” [42]	
	Access to and quality of green open space	“The environment used was seen as a good walking experience because of the wide open spaces. This enabled participants to stride out ahead of others and personalise their walks as they saw fit. *'Out walking by the sea or in the country, it takes on a different atmosphere and focus and also you don’t realise while you are taking in the scenery just how far you are walking.'*” [54] *“(To encourage me to walk) you can refurbish the park. (...) We’ve got a park (Ward End Park), but it could be improved. We don’t have a (high quality) park!”* [30]	
	Access to older adult oriented activities	*“If I’ve got somewhere to go I’ll go. But I’ve nowhere to go, what can I do? I got nothing to do so I watch television... nobody offers anything around here.”* [31] *“If there was a community center for people over 65, I’d go out maybe every day”* [30]	
***Access to daily destinations***	Access to shops and services	*“We're lucky—the health centre's only down the road in –. Got a lovely library, you know, I do love the library.”* [53] *“I pop to the shop for bits if I need to, and oh… to the bookies to put a bet on the horses… I take each day as it comes, you know, I do alright I suppose.”* [38]	[27–31, 36, 38, 39, 45, 46, 51, 53]
	Access to public transit	*“Sometimes the bus times don’t suit you because they only run every hour”* [28] “Mary and Bill are in their early eighties and live independently in Brynithel, a village at the top of the Abertillery valley. Neither is able to drive and they report public transport to be particularly problematic as buses run infrequently and not directly to places they may need to access. This is a real concern in the winter months and after dark.” [39]	
	Access to benches and public toilets	*“if [older people] are out round to the shops, or the community centre here, they could always walk back and sit in there in the summer for half an hour if you like and have a rest. You have always got to remember that the older ones like us, you can get tired.”* [27] *“If I want to do more (walking), I would need more places where I can rest and more toilets”* [29]	
	Destination diversity	*“Long ago … Everything you needed was in Govan. There was numerous fish shops, butchers, fruit shops, shoe shops, various fashion shops, furniture shops, everything … You could come down to Govan on a Saturday and spend the afternoon in Govan. You can't now. Ten minutes and [claps] you've seen it.”* [51] “Local services were also few, and interviewees often said they didn’t go around the estate much, because they had *'no reason to'*.” [27]	
***Environmental quality***	Air pollution	*“I like round [XXXX] bridge. I like places like that. You know, there’s good fresh air and you meet other people.”* [47] *“I don't particularly like to walk on the main roads, for the simple reason of the traffic. Not that I'm afraid that I'll get knocked down. But it's the fumes. I can smell the diesel and it's not fresh air […]”* [53]	[27, 29, 35, 42, 45, 47, 53]
	Noise pollution	*“Last year, we'd a big burst water main at the Cross, so the traffic was all diverted. It was so peaceful, that, you know, it suddenly brought it home to you how much noise you were taking in every day. It was like being out in the country […] And you just felt, oh, I could walk more here, you know?”* [53] *“This is the area that I don’t like to walk. As I told you, there is a lot of noise”* [29]	
***Aesthetics***	Natural scenery	*“I will walk more if they (authorities) put some flowers and something like that. I will be quite happy with things like that”* [29] *“Well all the trees. There’s trees and nature. You see ducks and you see... So you go up there and you see ducks, and cows. Just trees. It’s not vandalised, not wrecked and ruined and destroyed. You know what I mean? So when you go up to the [XXXX] bridge, 10 min away in the car, sure it’s like a different world isn’t it?”* [47]	[27, 29, 30, 35, 42, 45–47, 51, 53, 54]
	Buildings and streetscape	*“They've built a new park and new houses, the Gorbals Park, which is lovely.”* [53] *“It (my neighbourhood) is boring. These little industries are around. There is not many pretty gardens and places to look up regularly. You can see all industries over there! Mixtures of old and new are round here, just the manufactory and industry, and what we call'back to back houses'!”* [29]	
	Traffic volume	*“Much better in this part I live in because I'm off the main road […] – Street, which is out the back, is very congested, but it's a lot quieter here.”* [53] *“I confess I do go outside my house, but only in the quiet times. I look very carefully (for crossing the roads). I can say I stop half way, on the white lines you see in the middle of the road”* [29]	

*SES* socio-economic status

The theme “safety” comprised two sub-themes: (1) traffic-related safety; and (2) crime-related safety. Traffic-related safety concerns included reckless driving, bicycles or vehicles on pavements, and difficulties crossing roads due to dangerous junctions and excessive speed. Crime and anti-social behaviour posed barriers to physical activity, especially for women. Some older adults felt intimidated by young people in parks, particularly if public spaces lacked a sense of collective ownership. On the other hand, features of supportive physical activity environments included adequate street lighting and camera surveillance systems. Furthermore, several aspects of the built environment or natural topography related to the theme of “pedestrian infrastructure” deterred physical activity. Sub-themes included: (1) quality and maintenance; (2) slopes and curbs; and (3) road crossings. The poor condition of pavements was evidenced by reports of uneven slabs, high curbs, and the presence of manholes, cracks, or obstacles. In addition to a lack of crossings at close intervals, these attributes restricted outdoor walking for participants with limited mobility.

“Access to physical activity opportunities” was sub-divided into: (1) access to recreational facilities; (2) walking and cycling infrastructure; (3) access to and quality of green open space; and (4) access to older adult oriented activities. Lack of access to local recreational facilities inhibited older adults’ physical activity participation. Walking and cycling infrastructure, including the presence of alternative routes and shortcuts, encouraged older adults to get to their destinations via active travel. While the availability of green spaces was important, these were often considered of low quality. The provision of age-appropriate exercise classes and groups was associated with socialisation and enjoyment. However, there were not enough perceived opportunities for community engagement in deprived areas.

In relation to the theme “access to daily destinations”, the following sub-themes were identified: (1) access to shops and services; (2) access to public transit; (3) access to benches and public toilets; and (4) destination diversity. Participants described local shops and services as incentives for physical activity (e.g., undertaking errands). Nonetheless, the presence of shops was not a sufficient reason to use them if they were perceived as undesirable or unaffordable. Public transit supported people to access their preferred walking routes further afield. In addition, older adults discussed lack of benches and public toilets as barriers to taking long walks. Notably, benches provided places to rest, whereas toilets gave participants the confidence to leave the house for extended periods of time. Public toilets in deprived areas were largely vandalised, and self-contained toilets were complicated to use, particularly for those with low literacy. The absence of different types of destinations in the neighbourhood was also seen as hindering physical activity.

The theme “environmental quality” consisted of: (1) air pollution; and (2) noise pollution. Specifically, older adults were more inclined to walk in peaceful neighbourhoods, with good fresh air, and low traffic density. Exposure to noise and air pollution (e.g., petrol fumes) dissuaded them from going out. The final theme, “aesthetics”, included the sub-themes: (1) natural scenery; (2) buildings and streetscape; and (3) traffic volume. Nature promoted physical activity, though green spaces in deprived neighbourhoods were often neglected. Poor maintenance and landscaping made walking unappealing, whereas low levels of physical disorder created a more inviting setting for physical activity. Participants disliked unattractive scenery, such as industries and motorways, indicating that clean, interesting streets, with low traffic volume and architecturally varied buildings would motivate them to be more active.

##### Social opportunity

Table 3 shows the factors influencing low-SES older adults’ social opportunity to be active. Eminent themes included: (1) social support; (2) social norms; (3) social engagement; and (4) dog ownership.

**Table 3:** Factors influencing low-SES older adults’ social opportunity to engage in physical activity

**Themes**	**Sub-themes**	**Extracts and/or quotes**	**Contributing studies**
***Social support***	Emotional	*“I think it is loneliness from my point of view. Because people found out about you…When you are not going to the rehab classes, you are left to your own devices, and if you can’t get motivated to go to do the exercises, you need somebody, the company to get you going…”* [41] *“I’m a very lazy person in the sense that I need people around me to push me to do exercises, and I find it better when there’s a crowd of you, you know. Now if my two exercise partners don’t want to go to the gym where I live now, I don’t go. And I get annoyed with myself then …”* [34]	[27, 28, 32–34, 36, 38, 39, 41, 43, 46–49, 52, 54, 55]
	Practical	*“... if I need to go somewhere, to the hospital for an appointment to the clinic, my daughters are down straight away. Pick me up in the car, so, I don’t get to walk, and I would, and that, that would stop me from, you know.”* [47] “Emily is in her seventies and lives in a supported housing complex in Llanhilleth. Her mobility is extremely limited and she is unable to get out and about as easily as she once used to, now relying upon family and public transport.” [39]	
	Informational	*“Well I was encouraged, but not pushed... So it’s obviously a lot better than virtually being told, what about this or no sorry, do this or this or this. So it was open and it enabled me to do it at the pace I wanted it to.”* [43] *“I found that she ('Sarah') was a link to me doing what I wanted to do which was this keep fit (exercise referral) class, getting out, meeting people.”* [48]	
***Social norms***	Social comparison	*“When I started the exercising it was a one on one, then [a healthy living center member] started, it was better fun than when there was one person, because you encouraged each other on, a wee bit of friendly competition bit of a laugh and all”* [28] *“Yes, I’ve started walking faster. I used to be at the back and now I’m in the middle and I want to be right up the front.”* [54]	[28, 31–33, 36, 38, 39, 41, 43, 47, 48, 51, 52, 54]
	Physical activity norms	“Further, activities traditionally considered healthy were often perceived as alien and even a source of amusement by some older adults: Researcher: *'I mentioned I'm interested in health, so when I talk about health It's like what you eat, exercise, diet. What's your views on that? Just general?'* Vincent: *'Well, for me first thing in the morning, do 24 press ups (laughs) … And he [points at another person] can just about walk to that door! (laughs) And he just uses it now for fun, he could get up and run around if he can. (laughs). But no, we're not very good but you get by.'*” [38] *“People have said to me,'Oh... you’ve done Sheffield round walk – which is about 16 mile – you must be daft!'”* [43]	
***Social engagement***	Participation	“Clive: *'Well I take me dog out about half past four in the morning… Cause’ I’ve got a dog… Rocky…. I go down there up Burton Street, across where them lights are on the lift then under the subway and back … Then I come to work here upstairs for about what an hour, an hour and a half.'* For Clive, his daily practices always involved walking his dog at specific times and visiting the community centre to help out and have a meal. Importantly, visiting and helping in the community centre were also occasions to socialise with friends and acquaintances.” [38] “She is unhappy with the lack of social activities at the housing complex and the fact that the residents are not encouraged to socialise.” [39]	[27, 31, 33, 35, 36, 38, 39, 41, 44, 46, 51, 54]
	Identity	*“So you get to be anti-social... removed from people and activities and things like that. It’s not good for the health... they don’t come to tell me anymore things like'oh there’s this party going on, are you gonna come?'”* [31] *“You would be talking to the other people (at CR) and we’re all in the same boat. I felt it built up your confidence and get fitter.”* [41]	
***Dog ownership***		*“It is really good. I am more active now than I was three or four years ago. I just go on walks not take my car. Take the dog out four or five times a day.”* [48] *“I think I’m relatively inactive actually. … We … lost our dog, which was always a perfect excuse to have to go out to take the dog for a walk, and my partner used to be very active as far as walking was concerned and now that’s gone [since being wheelchair bound]. The motivation isn’t there, I don’t feel motivated so much to do things physically on my own…”* [44]	[32, 34–36, 38, 44, 47, 48]

*SES* socio-economic status, *CR* cardiac rehabilitation

The theme “social support” was divided into three sub-themes: (1) emotional; (2) practical; and (3) informational. Emotional support was most frequently mentioned. Social interaction improved enjoyment, reduced isolation, increased confidence, and provided a distraction from the strain of physical activity. However, some older adults had trouble identifying a suitable “buddy”. Practical support involved relying on family and friends to provide transport. Both lack of support and too much support, where participants were regularly driven to destinations rather than walking, impeded behaviour change. While informational support was seen as less important, older adults who had taken part in a behavioural intervention still desired feedback on their physical activity levels. Information was also sought on exercise opportunities and alternative physical activity strategies.

“Social norms” was represented by the following sub-themes: (1) social comparison; and (2) physical activity norms. Older adults described a sense of relatedness when exercising alongside like-minded people, although certain activities (e.g., swimming) were perceived as inapplicable to their age group. Social comparison acted to improve physical activity intensity, while age-aligned role models fostered accountability. A few participants’ family members (e.g., spouses) had inspired them to make lifestyle modifications.

The “social engagement” theme touched on: (1) participation; and (2) identity. Older adults referred to different forms of social participation as enablers of physical activity, including voluntary work, caring responsibilities, club memberships, and community centre involvement. While a sense of group identity facilitated physical activity, individuals who felt disconnected from their communities engaged in more sedentary pursuits. Finally, “dog ownership” was a source of motivation, which supported older adults to walk as part of their daily routines. Some lost an “excuse” to be active when their dog(s) passed away.

#### Motivation

An overview of the themes and illustrating quotes for the motivation component of the COM-B model is provided in Table 4.

**Table 4:** Factors influencing low-SES older adults’ motivation to engage in physical activity

**COM-B sub-component**	**Themes**	**Extracts and/or quotes**	**Contributing studies**
***Reflective motivation***	Outcome expectancies	*“It (PA) has definitely changed my life. It has lifted the depression big time”* [28] *“Yes, I do a lot, because I’m doing them exercises it’s helped me, it’s good for my health, I feel much better, I can breathe properly. And you make friend. Yeah, it’s good for me—I go out, and you meet friends.”* [55]	[27, 28, 32, 34, 36, 38, 39, 41–44, 46–50, 54, 55]
	Self-efficacy	*“These (walking) boots I bought, 3 or 4 year ago and they’ve sat on a shelf 2 years. I haven’t had a chance to go walking on me own…now have confidence to walk on my own.”* [54]	[34, 41, 50, 54]
	Attitudes	“George considers free swimming to be a *'waste of time.'* He felt most older people can’t swim and it is really too late to try and learn as many people have health problems and are not interested.” [39] *“To anybody that doesn’t walk and they’re quite capable... I find that as alien as what they find me doing them sort of things, not doing anything when you’re quite capable that you could do something.”* [43]	[32, 33, 36, 39, 41, 43, 44, 49]
***Automatic motivation***	Habits and routines	*“It is a regime and you’ve got dedicated times makes me more inclined to go the fixed regime that you have a walk at this time.”* [54] *“Laziness really, once I got out walking I was grand. It was just getting out of that habit of sitting on the sofa and once you got out it was good and when you came back you felt brilliant that you had got out and you had achieved what you were looking for.”* [47]	[32–34, 36, 38, 39, 42–44, 46, 47, 50, 54]
	Lack of time	*“Being at work you’ve got to squeeze physical activity in whereas when I will be retired it will be'ah I got time to do that'”* [33] *“Cos I do still do some part time work – I look after a lady with dementia – so I’m not free every day by any means. So the main thing is, sort of fitting it in around... Once a week is fine. Fitting in twice or three times can be difficult sometimes to find that bit of free time.”* [43]	[32–34, 36, 39, 43, 44, 47]
	Enjoyment and self-identity	*“No I think it helps that when you enjoy it, you wouldn’t be doing it otherwise. It’s a big factor”* [28] *“I find it more difficult to keep motivated, cos I don’t really like exercise. I could... I’m one of these people that could just sit and watch telly all day and enjoy it.”* [43]	[28, 32, 34, 36, 38, 39, 41–44, 46, 47, 54]

*SES* socio-economic status, *COM-B* Capability, Opportunity, Motivation, and Behaviour, *PA* physical activity

##### Reflective motivation

The following three themes were grouped under reflective motivation: (1) outcome expectancies; (2) self-efficacy; and (3) attitudes. Many older adults were motivated by positive outcome expectancies, expressing beliefs that physical activity could alleviate symptoms of existing conditions, prevent new ones from developing, promote longevity, improve physical function, assist with fitness or weight loss goals, increase confidence, enhance wellbeing, and boost networks. For some, physical activity was deemed worthwhile only if it served a purpose (e.g., as a mode of transport). High self-efficacy was a physical activity enabler, which older adults built and strengthened by preparing to exercise in unfamiliar settings, understanding the levels of physical activity they should and could undertake safely, and normalising exercise as an essential part of life. Older adults who exhibited disinterest in physical activity discussed competing priorities as a barrier to participation. In contrast, those who embraced physical activity as part of their identity made an intrinsic commitment which supported long-term behavioural change.

##### Automatic motivation

Automatic motivation was characterised by the themes: (1) habits and routines; (2) lack of time; as well as (3) enjoyment and self-identity. Physical activity habits were often established at youth and internalised as a way of life. However, events such as retirement or the cessation of exercise programmes compelled older adults to develop new routines. Engaging in organised physical activity reinforced automatic motivation during these transitions. Social support encouraged older adults to break sedentary habits and fostered sustained involvement in physical activity. For those who lacked previous positive experiences of physical activity or exercise, habits were born out of more extrinsic motives. Few older adults made references to lack of time as a barrier; this was mainly confined to individuals in part- or full-time work, and those caring for partners or grandchildren. Enjoyment was an important facilitator of physical activity, which was associated with social company and specific activity types. Positive emotions and sensations were felt by older adults who described themselves as sporty, whereas rejecting physical activity as part of one’s self-identity was seemingly associated with a propensity for sedentary alternatives, such as watching television.

### Enablers and barriers among older adults of low versus high SES

Table 5 summarises findings from the five studies examining inequalities in physical activity enablers and barriers among older adults. The studies contributed primarily to the physical opportunity (*N* = 5) and social opportunity (*N* = 3) COM-B sub-components. Differences in the salience of enablers and barriers across the reflective and automatic motivation sub-components were not identified or presented in these studies. One study found that health conditions encouraged a change in attitude towards physical activity in the high-SES group, whereas they kept low-SES older adults from participating in physical activity altogether. High-SES older adults had superior knowledge of the physical activity guidelines relative to their low-SES counterparts. Neighbourhood safety was a more prominent concern in low-SES individuals, who reported a higher presence of traffic hazards, poor visibility, and anti-social behaviour. While low-SES older adults remarked on a lack of opportunities for community engagement, high-SES older adults generally had access to a variety of clubs and groups, as well as spaces or amenities that facilitated physical activity behaviour, including benches to rest on and public toilets. Considerable disparities were identified in environmental quality and the attractiveness of the natural and built environment, which favoured high-SES older adults, and may have contributed to a greater sense of belonging in their communities.

**Table 5:** Inequalities in physical activity enablers and barriers among older adults

**Low SES**	**High SES**	**Contributing studies**
**Capability**		
***Physical capability*** a) General health ▪ Health conditions cited as a barrier to physical activity	***Physical capability*** a) General health ▪ Health conditions cited as an enabler of physical activity	[28]
***Psychological capability*** a) Knowledge and thought ▪ Limited knowledge of the current physical activity guidelines	***Psychological capability*** a) Knowledge and thought ▪ Comprehensive knowledge of the current physical activity guidelines	[28]
**Opportunity (physical)**		
***Safety*** a) Traffic-related safety ▪ Higher presence of traffic hazards (e.g., high speed, lack of traffic lights, presence of cars/lorries on pavements)	***Safety*** a) Traffic-related safety ▪ Good visibility offered a sense of protection against certain traffic hazards ▪ Traffic hazards mainly related to the presence of schools (e.g., children being brought to school by car)	[27–31]
b) Crime-related safety ▪ Lack of safety, particularly for women ▪ Presence of gangs and anti-social behaviour (i.e., felt vulnerable to violence, particularly regarding young people) ▪ Lack of infrastructure to improve safety (e.g., poor visibility)	b) Crime-related safety ▪ Perceived safety in their neighbourhoods ▪ No real crime or “frightening” people ▪ Infrastructure to improve safety (e.g., streetlights)	
***Pedestrian infrastructure*** a) Quality and maintenance/Slopes and curbs ▪ Goods outside shops made pavements narrow ▪ More issues related to uneven pavements, broken slabs, presence of potholes, and cracks	***Pedestrian infrastructure*** a) Quality and maintenance/Slopes and curbs ▪ Trees/bushes/hedges from adjacent gardens and disturbance from infrastructure repairs made pavements narrow ▪ More issues related to high curbs and cluttered streets	[27–29]
b) Road crossings ▪ Lack of crossings (especially at close intervals)	b) Road crossings ▪ Easier to cross the roads (as a by-product of lower traffic density)	
***Access to physical activity opportunities*** a) Access to recreational facilities/Access to older adult oriented activities ▪ Lack of recreation centres or opportunities for community engagement	***Access to physical activity opportunities*** a) Access to recreational facilities/Access to older adult oriented activities ▪ Variety and choice of physical activities, sports, clubs, and groups	[27–31]
b) Access to and quality of green open space ▪ Low-quality (i.e., small, dirty, unsafe) parks and green open spaces	b) Access to and quality of green open space ▪ High-quality (i.e., large, attractive, safe) parks and green open spaces	
***Access to daily destinations*** a) Access to shops and services ▪ Lack of (attractive) local services for socialising	***Access to daily destinations*** a) Access to shops and services ▪ Availability of diverse destinations (e.g., cafés) to meet and socialise	[27–31]
b) Access to benches and public toilets ▪ Too few benches to rest on (or they were unsafe/vandalised) ▪ Public toilets closed due to safety issues (e.g., vandalism) ▪ Pay toilets perceived as not user-friendly	b) Access to benches and public toilets ▪ Plenty of benches to sit and rest on ▪ Safety and user-friendliness of public toilets less of an issue	
***Environmental quality*** a) Air pollution ▪ Poor air quality (as a by-product of high traffic density)	***Environmental quality*** a) Air pollution ▪ Good air quality (as a by-product of low traffic density)	[27, 29]
b) Noise pollution ▪ Annoyance about noise (i.e., from traffic) in their neighbourhoods	b) Noise pollution ▪ Satisfaction with quietness of their neighbourhoods	
***Aesthetics*** a) Natural scenery ▪ Lack of greenery and trees ▪ Poor maintenance and landscaping of the local area ▪ Small, empty grass-covered spaces lacking vegetation (parks in need of refurbishment) ▪ Overgrown green spaces, littered with broken glass	***Aesthetics*** a) Natural scenery ▪ Presence of greenery ▪ Nice natural landscapes nearby ▪ Large, historical, and beautiful green spaces (as well as good overall cleanliness) ▪ Well-maintained gardens and flower beds	[27, 29, 30]
b) Buildings and streetscape ▪ Lack of attractive houses and buildings (as well as dirty streets/alleys) ▪ Presence of many industrial sites in their neighbourhoods (and previous green spaces built on by recent housing developments)	b) Buildings and streetscape ▪ Attractive buildings and commercial areas ▪ No or few industries in their neighbourhoods	
c) Traffic volume ▪ High traffic density (as a physical barrier to reaching destinations)	c) Traffic volume ▪ Low traffic density	
**Opportunity (social)**		
***Social engagement*** a) Identity ▪ Felt a sense of disconnection in their communities	***Social engagement*** a) Identity ▪ Felt a sense of belonging in their communities	[27, 31]

*SES* socio-economic status

## Discussion

This meta-synthesis of thirty studies utilising qualitative methods sought to identify the modifiable enablers of, and barriers to, physical activity behaviour among low-SES older adults in the UK. A large proportion of factors derived from the available evidence focused on physical (e.g., safety, access to facilities, neighbourhood aesthetics) and social (e.g., social support, norms) opportunity, as well as reflective motivation (e.g., outcome expectancies). Half of the studies presented detail on the role of physical capability (e.g., mobility, general health) and automatic motivation (e.g., habits, enjoyment), with a few studies homing in on psychological capability (e.g., knowledge and behavioural regulation). Using the COM-B model as a framework for categorising these enablers and barriers, we have shown that in the eyes of older adults, physical activity is driven by a wide range of, often interacting, factors, reinforcing the complex nature of this health behaviour [15, 18, 57]. Very few studies examined nor presented data on differences between participants of low and high SES, with those that did predominantly pointing to disparities in the physical environment as enabling or inhibiting physical activity respectively.

The emphasis on environmental factors, categorised under the physical opportunity sub-component of the COM-B model, is congruent with the concurrent quantitative systematic review with meta-analysis that found variables such as the amount of green space, built or natural spaces for physical activity, and walking or cycling infrastructure to be widely studied [17]. Except for the presence of built physical activity facilities or walking and cycling infrastructure, which were deemed in the meta-analysis to be positively associated with physical activity in low- but not high-SES older adults (although the confidence intervals from the respective stratified meta-analyses in low versus high-SES older adults overlapped), there was little difference in the direction (i.e., positive or negative) or magnitude of the associations of these variables with physical activity behaviour, a pattern that was consistent across the capability and social opportunity related factors. The findings of the present review give credence to the assertion made by Malkowski and colleagues [17] that it is likely the prevalence in which different social strata are exposed to favourable and less favourable intrapersonal, interpersonal, or environmental conditions that explains inequalities rather than differences in the way those conditions influence physical activity behaviour. Of note, low-SES older adults included in this meta-synthesis of qualitative literature perceived access and proximity to parks or walking routes as inadequate for inciting behaviour change, if crime, hazardous pedestrian infrastructure, and/or lack of beauty persisted, suggesting the positive influences of these spaces for promoting physical activity are not solely attributable to their quantities, but rather to their qualities [58].

Several factors identified in this qualitative meta-synthesis were not assessed in enough studies to be meta-analysed in the concurrent review of quantitative evidence. A stark difference is the presence of motivational factors that were seldom captured in the quantitative review but came through in a large proportion here and have been shown to be important for older adults’ physical activity behaviour [12, 59]. This may be explained by different study designs and objectives within the papers from which the data feeding into the reviews were derived. For example, large quantitative cohort studies addressing research questions on the dynamics of ageing or disease, and which do not explicitly focus on physical activity, are unlikely to include measures of motivational processes specific to a given health behaviour. Moreover, the qualitative synthesis affords novel insights into the interacting nature of the identified enablers and barriers. The COM-B model posits that Capability, Opportunity, and Motivation do not influence behaviour in isolation [10], and the qualitative data in this review illustrate this point among low-SES older adults. For example, poor fitness and mobility (physical capability) were discussed in the context of there being insufficient leisure provision accounting for these needs (physical opportunity), resulting in a lack of physical activity enjoyment (motivation) and a necessity of familial help (social support). These findings highlight the value of mixed methods research in painting a full picture of behaviour, as the dynamic interrelations that underpin the socio-ecological and COM-B models could not be evaluated meta-analytically and would have been overlooked if we only took the separate quantitative findings into account to inform the necessary ingredients of behavioural interventions for this population.

Indeed, the complex interplay of factors that seem important for narrowing the SES divide in older adults’ physical activity behaviour reinforces calls for policy makers and practitioners in the UK to consider solutions targeting all levels of the socio-ecological model [57]. At the wider societal level, policies prioritising clean, safe, and aesthetically pleasing environments for physical activity, with places to rest, are required. Beyond more equitable investment to repair and improve existing infrastructure in disadvantaged neighbourhoods, the ‘15-min city’ initiative, an urban planning concept which intends to give people access to local amenities and services with hyper proximity, appears promising in this context [60]. This would also support participants who described physical activity as a by-product of heightened community engagement. At the interpersonal level, interventions that facilitate social support and peer-modelling of physical activity may be beneficial for low-SES older adults. The age-inclusive REtirement in ACTion [61] and peer support focused Active, Connected, Engaged [62] interventions are cost-effective examples in the UK that might be well-suited to deprived localities. Within this, it would also be vital to propose enjoyable, affordable options that meet the needs of people with mobility impairment. The systems approach is therefore particularly apt for ensuring that any initial efforts to change behaviour by low-SES older adults can be sustained through continued reinforcement of the social and physical environments.

The present study offers unique insights into the multitude of factors that influence physical activity among UK-based, community-dwelling, low-SES older adults, derived from comprehensive literature searches, rigorous extraction processes, and contemporary behaviour change frameworks. There are, however, several limitations that warrant consideration when interpreting the findings. First, the review focuses on the UK setting and so may not be indicative of other countries. Indeed, physical activity preferences vary across different settings, and while some of our findings may be generalisable to other countries, policy responses should be adapted to local contexts to cultivate socially and culturally appropriate experiences [19]. Secondly, the findings are confined to the themes and quotes presented by the original study authors, as we did not interrogate raw transcripts. While this is common practice in systematic reviews of qualitative literature, we may have missed relevant participant experiences or perceptions that only pertained to a minority of the sample or were not chosen to illustrate the original study authors’ interpretations. As appropriate and practical solutions to sharing qualitative data become available, new opportunities for more in-depth secondary data analyses of open qualitative data could arise. Moreover, the research questions in the included studies did not always map directly onto the aim of the current review, with few studies contrasting individuals of low and high SES. Accordingly, the large volume of data addressing enablers and barriers pertaining to the physical opportunity sub-component of the COM-B model, compared to the automatic motivation sub-component for instance, may reflect a heavier focus on the built or natural environment within interview topic guides rather than its relative importance. Thirdly, some of the qualitative results stemmed from process evaluations of intervention studies, which may pose an inherent bias towards positive appraisals in response to successful programmes or recent, atypical, physical activity participation.

## Conclusions

Notwithstanding these limitations, this is the first study to synthesise qualitative literature on the enablers of, and barriers to, physical activity behaviour among low-SES older adults in the UK. The findings not only corroborate our comprehensive quantitative review in implying that the prevalence or experience rather than the type of influences may be driving inequalities, but also add to our knowledge of the complex interacting mechanisms underpinning physical activity in this demographic. Further empirical studies could report results by SES to enhance our understanding of inequalities and ensure that low-SES older adults are represented in the development and evaluation of interventions targeting improvements in physical activity by using co-creation, co-design, or co-production approaches. Moreover, whole-system interventions that recognise interrelationships across socio-ecological and COM-B domains are likely to be necessary for meaningful and sustained change that contributes to reduced health inequalities in later life.

## Supplementary Information

Additional file 1. PRISMA guidelines.

Additional file 2. Search strategy for each database.

Additional file 3. Data extraction form.

Additional file 4. Mixed Methods Appraisal Tool (MMAT) guidance notes.

Additional file 5. Characteristics of the included studies.

## Data Availability

All data generated or analysed during this study are included in this published article and its additional files.

## Abbreviations

SES: Socio-economic status
UK: United Kingdom
CENTRAL: Cochrane Central Register of Controlled Trials
MMAT: Mixed Methods Appraisal Tool
ISRCTN: International Standard Randomised Controlled Trial Number
PRISMA: Preferred Reporting Items for Systematic Reviews and Meta-Analyses
PROSPERO: International prospective register of systematic reviews
COM-B: Capability, Opportunity, Motivation, and Behaviour
