# Supplementary material for: Enablers and barriers to physical activity among older adults of low socio-economic status: a systematic review of qualitative literature
Source: Int J Behav Nutr Phys Act. 2025 Jun 23;22:82. doi: 10.1186/s12966-025-01753-4 (PMC12183869; doi:10.1186/s12966-025-01753-4)
Supplement: Supplementary file 3 — Additional file 3. Data extraction form. [file 12966_2025_1753_MOESM3_ESM.docx]

**Additional file 3.** Data extraction form

| **General information** |
| --- |
| **Study ID**  ……………………………… |
| **Study citation**  *Study citation (can be copied in format reported in publication and pasted here directly)*  ……………………………… |
| **Lead and/or corresponding author contact details**  *Email address*  ……………………………… |
| **Study aims**  ……………………………… |
| **Study design**  Randomised controlled trial  Non-randomised experimental study  Baseline from randomised controlled trial  Baseline from non-randomised experimental study  Prospective study  Repeated cross-sectional study  Cross-sectional study  Qualitative research  Other  ……………………………… |
| **Include/Exclude**  *All papers at this stage should normally be "included" as they have been checked for eligibility at the full-text screening stage. This is just confirmation of the decision.*  Include  Exclude |
| **Reason for exclusion**  ……………………………… |
| **Reporting guidelines**  *Mention whether or not a reporting guideline/checklist was used and name it (e.g., CONSORT, STROBE).*  ……………………………… |
| **Data collection** |
| **Start date (of data collection, or wave/year for prospective studies)**  ……………………………… |
| **End date (of data collection, or wave/year for prospective studies)**  ……………………………… |
| **Participants and setting** |
| **Population (observational/qualitative/mixed-methods studies)** |
| **Population description**  ……………………………… |
| **Inclusion criteria (of the study)**  ……………………………… |
| **Exclusion criteria (of the study)**  ……………………………… |
| **Total number of participants (and sample size in analyses of interest if different, including stratified by socioeconomic group)**  ……………………………… |
| **Sampling method and stratification**  ……………………………… |
| **Age, range and mean – for total sample and sample in analyses of interest (total and stratified by socioeconomic group) if different**  ……………………………… |
| **Sex (% female) – for total sample and sample in analyses of interest (total and stratified by socioeconomic group) if different**  ……………………………… |
| **Ethnicity**  ……………………………… |
| **Socioeconomic status (include details of indicator/measure as well as descriptive statistics)**  ……………………………… |
| **Pathology**  ……………………………… |
| **Other relevant baseline descriptive characteristics**   \|  \| ***Total number of participants*** \| ***Sample size in analyses of interest*** \| ***Low socioeconomic group*** \| ***High socioeconomic group*** \| \| --- \| --- \| --- \| --- \| --- \| \| Characteristic 1 \|  \|  \|  \|  \| \| Characteristic 2 \|  \|  \|  \|  \| \| Characteristic 3 \|  \|  \|  \|  \| \| Characteristic 4 \|  \|  \|  \|  \| |
| **Population (intervention)** |
| **Population description**  ……………………………… |
| **Inclusion criteria (of the study)**  ……………………………… |
| **Exclusion criteria (of the study)**  ……………………………… |
| **Total number of participants (and sample size in analyses of interest if different, including stratified by socioeconomic group)**  ……………………………… |
| **Sampling method and stratification**  ……………………………… |
| **Age, range and mean – for total sample and sample in analyses of interest (total and stratified by socioeconomic group) if different**  ……………………………… |
| **Sex (% female) – for total sample and sample in analyses of interest (total and stratified by socioeconomic group) if different**  ……………………………… |
| **Ethnicity**  ……………………………… |
| **Socioeconomic status (include details of indicator/measure as well as descriptive statistics)**  ……………………………… |
| **Pathology**  ……………………………… |
| **Population (comparator)** |
| **Population description**  ……………………………… |
| **Inclusion criteria (of the study)**  *Please ignore if already filled out for intervention section (unless different)*  ……………………………… |
| **Exclusion criteria (of the study)**  *Please ignore if already filled out for intervention section (unless different)*  ……………………………… |
| **Total number of participants (and sample size in analyses of interest if different, including stratified by socioeconomic group)**  ……………………………… |
| **Sampling method and stratification**  ……………………………… |
| **Age, range and mean – for total sample and sample in analyses of interest (total and stratified by socioeconomic group) if different**  ……………………………… |
| **Sex (% female) – for total sample and sample in analyses of interest (total and stratified by socioeconomic group) if different**  ……………………………… |
| **Ethnicity**  ……………………………… |
| **Socioeconomic status (include details of indicator/measure as well as descriptive statistics)**  ……………………………… |
| **Pathology**  ……………………………… |
| **Population (intervention/comparator)** |
| **Other relevant baseline descriptive statistics**   \|  \| ***Intervention***  ***(total number of participants)*** \| ***Comparator***  ***(total number of participants)*** \| ***Intervention***  ***(sample size in analyses of interest)*** \| ***Comparator***  ***(sample size in analyses of interest)*** \| ***High socioeconomic group***  ***(overall)*** \| ***Low socioeconomic group***  ***(overall)*** \| ***High socioeconomic group***  ***(intervention)*** \| ***Low socioeconomic group***  ***(intervention)*** \| ***High socioeconomic group***  ***(comparator)*** \| ***Low socioeconomic group***  ***(comparator)*** \| \| --- \| --- \| --- \| --- \| --- \| --- \| --- \| --- \| --- \| --- \| --- \| \| Characteristic 1 \|  \|  \|  \|  \|  \|  \|  \|  \|  \|  \| \| Characteristic 2 \|  \|  \|  \|  \|  \|  \|  \|  \|  \|  \| \| Characteristic 3 \|  \|  \|  \|  \|  \|  \|  \|  \|  \|  \| \| Characteristic 4 \|  \|  \|  \|  \|  \|  \|  \|  \|  \|  \| |
| **Setting** |
| **Context**  ……………………………… |
| **Location**  ……………………………… |
| **Nation/nations in the United Kingdom (UK)**  *Include detail of other countries investigated (for cross-national studies)*  ……………………………… |
| **Other relevant information**  ……………………………… |
| **Inclusion criteria (of the review)** |
| **All participants are aged ≥ 60 years (or data are stratified/reported by age group), or (for mixed populations where data are not stratified/reported by age group) the mean age of participants is ≥ 60 years and no participants are aged less than 50 years**  Yes  Yes, after receiving separate data from authors or extracting separate data from openly available datasets  No  Unclear |
| **All participants are community-dwelling (or data are stratified/reported by living situation)**  Yes  Yes, after receiving separate data from authors or extracting separate data from openly available datasets  No  Unclear |
| **Study conducted in the UK (or includes UK-based participants if online and data are stratified/reported by country)**  Yes  Yes, after receiving separate data from authors or extracting separate data from openly available datasets  No  Unclear |
| **Results categorised or reported (e.g., stratified) by socioeconomic status**  Yes  Yes, after receiving separate data from authors or extracting separate data from openly available datasets  No  Unclear |
| **Peer-reviewed**  Yes  No  Unclear |
| **Published in English**  Yes  No  Unclear |
| **Exclusion criteria (of the review)** |
| **Data retrieved from a conference abstract, dissertation, thesis, editorial, opinion, letter, trial protocol, review (e.g., systematic review, scoping review, meta-analysis), or case report**  Yes  No  Unclear |
| **Includes participants living in hospitals, assisted living accommodation, residential care homes, nursing homes, or any other institution (and disaggregated data on community-dwelling participants are not available)**  Yes  No  Unclear |
| **Correlates/determinants/facilitators (observational/qualitative/mixed-methods studies)** |
| **Study description**  ……………………………… |
| **Theoretical framework**  ……………………………… |
| **Methodology and methods**  ……………………………… |
| **Investigated exposures**  ……………………………… |
| **Measures of exposures**  ……………………………… |
| **Extracted quotes (qualitative)**  ……………………………… |
| **Length of follow-up**  ……………………………… |
| **Number of follow-up measurements**  ……………………………… |
| **Correlates/determinants/facilitators (intervention studies)** |
| **Intervention** |
| **Intervention description (content)**  ……………………………… |
| **Theoretical framework**  ……………………………… |
| **Methodology and methods**  ……………………………… |
| **Investigated exposures**  ……………………………… |
| **Measures of exposures**  ……………………………… |
| **Length of follow-up**  ……………………………… |
| **Number of follow-up measurements**  ……………………………… |
| **Proposed mediator(s)**  ……………………………… |
| **Any other information**  ……………………………… |
| **Comparator** |
| **Description (what was used by the control group)**  ……………………………… |
| **Investigated exposures**  ……………………………… |
| **Measures of exposures**  ……………………………… |
| **Outcome/Evaluation** |
| **Primary outcome** |
| **Primary outcome (e.g., unstructured physical activity, structured exercise, or a combination of physical activity and exercise)**  *Please choose one from the list above (there is an opportunity to expand in the next section)*  ……………………………… |
| **Primary measure/operationalisation**  ……………………………… |
| **Results for each correlate, determinant, and/or facilitator: Positive association (+), negative association (-), no association (NS)***  **For quantitative studies, associations are based on statistical significance*  *Note: Please ignore this section if exposure/outcome data were not extracted directly from the publication (i.e., separate data were obtained by contacting study authors or extracting from openly available datasets)*  ……………………………… |
| **Attrition rate**  *Please ignore for cross-sectional studies*  ……………………………… |
| **Statistical method(s) used**  ……………………………… |
| **Covariates**  *Please ignore if variable of interest was itself a covariate in the original analyses*  *Please only list covariates if variable of interest corresponds to main exposure in the original analyses*  ……………………………… |
| **Moderators**  *Please only list moderators if variable of interest corresponds to main exposure in the original analyses*  ……………………………… |
| **Effect size**  ……………………………… |
| **Qualitative data analysis**  ……………………………… |
| **Other outcomes** |
| **Other outcomes**  ……………………………… |
| **Measures/operationalisations**  ……………………………… |
| **Results**  ……………………………… |
| **Statistical method(s) used**  ……………………………… |
| **Effect size: main effects or moderating effects**  ……………………………… |
| **Qualitative data analysis**  ……………………………… |
| **Other** |
| **Ethical approval**  *Ethical approval statement from publication*  ……………………………… |
| **Limitations (author)**  ……………………………… |
| **Limitations (reviewer)**  ……………………………… |
| **Conclusions (author)**  ……………………………… |
| **Important notes for the assessment or interpretation of the study (if any)**  ……………………………… |
| **Study funding sources**  *Funding statement from publication*  ……………………………… |
| **Possible conflicts of interest for study authors**  *Conflicts of interest statement from publication*  ……………………………… |
