# Supplementary material for: Enablers and barriers to physical activity among older adults of low socio-economic status: a systematic review of qualitative literature
Source: Int J Behav Nutr Phys Act. 2025 Jun 23;22:82. doi: 10.1186/s12966-025-01753-4 (PMC12183869; doi:10.1186/s12966-025-01753-4)
Supplement: Supplementary file 5 — Additional file 5. Characteristics of the included studies. [file 12966_2025_1753_MOESM5_ESM.docx]

**Additional file 5.** Characteristics of the included studies

| **Author(s), year, and country** | **Primary aim(s) verbatim** | **Perspective/Theoretical framework^a^** | **Study design**  **Funding source(s)** | **Sample characteristics** | **Qualitative methods^b^** | **Data analysis** |
| --- | --- | --- | --- | --- | --- | --- |
| Anderson et al., 2014 [39]  Wales | The aim of this research was to explore the perceptions of non-users alongside the views of community leisure providers towards free swimming in an area of social and economic deprivation. | Not stated | Cross-sectional; qualitative  Not reported | 20 participants (age range: ≥ 60 years); 14 female, 6 male  SES (deprived wards):  Low: 20  High: 0 | Interviews | Thematic analysis |
| Barnett et al., 2013 [32]  England | This qualitative study aimed to explore and describe how cohabitating partners, of whom at least one has recently retired, influence each other’s physical activity behaviour. | Not stated | Cross-sectional; qualitative  British Heart Foundation Economic and Social Research Council  Medical Research Council  National Institute for Health Research  Wellcome Trust | 7 couples or 14 participants (age range: 60 to 70 years); 7 female, 7 male  SES (occupational class):  Low (manual): 4  High (non-manual): 10 | Interviews | Data-driven content analysis |
| Cavill and Watkins, 2007 [42]  England | The purpose of this paper is to explore views about cycling among members of identified community groups living near the Loop Line, a cycling and walking path in a deprived part of North Liverpool, UK. | Not stated | Cross-sectional; qualitative  Not reported | 23 participants (age range: ≥ 11 years); 14 female, 9 male  SES (deprived area):  Low: 23  High: 0  *Sub-sample:*  10 participants aged 50+ years (most 60 to 70 years); 5 female, 5 male  SES (deprived area):  Low: 10  High: 0 | Focus groups | Thematic analysis |
| Clark et al., 2005 [41]  Scotland | The aim of this paper is to report patients’ experiences of cardiac rehabilitation and perceptions of the mechanisms and contexts influencing its long-term effectiveness. | Realist approach | Cross-sectional; qualitative  ‘Have a Heart Paisley’, the Scottish Executive National Demonstration Project for Coronary Heart Disease | 47 participants (age range: 51 to 84 years; mean age 68.2 ± 8.2 years); 17 female, 30 male  SES (deprived region):  Low: 47  High: 0 | Focus groups | Framework analysis |
| Day, 2008 [27]  Scotland^c^ | Based on the perspectives of older people themselves in three urban neighbourhoods in the Glasgow region of Scotland, this article explores the ways in which the local outdoor physical environment may support or challenge older people’s health. | Not stated | Cross-sectional; qualitative  Not reported | 45 participants (age range: ≥ 62 years); at least 7 female, at least 3 male  SES (case study localities of low and high levels of deprivation according to the 2006 Scottish IMD):  Low (Inner City Neighbourhood/Suburban Estate): at least 6 participants with quotes  High (Small Coastal Town): at least 4 participants with quotes (and 2 couples) | Interviews (and field observation – not conducted with participants) | Thematic analysis |
| Derges et al., 2014 [55]  England | The primary aim of the qualitative study was to examine the causal pathways that generated any intervention effects from the perspectives of local residents, who were involved as strategic partners in Well London’s design and delivery. | Not stated | Prospective; qualitative  Wellcome Trust | Interview 1: 61 participants (age range: 16 to 75 years); 45 female, 16 male  SES (deprived neighbourhoods according to the 2010 English IMD):  Low: 61  High: 0  *Sub-sample:*  6 participants aged 66+ years; at least 1 female  SES (deprived neighbourhoods according to the 2010 English IMD):  Low: 6  High: 0  Interview 2: 45/61 participants | Interviews (nested within a cluster randomised trial) | Framework analysis |
| Forward et al., 2023 [50]  UK (not specified) | To explore the lived experience of living alone as an older woman (aged 65+) in the UK during the Covid pandemic. | Not stated | Cross-sectional; qualitative  Vice-Chancellor’s Scholarship from the University of West London | 7 participants (age range: 65 to 80 years); 7 female, 0 male  SES (education):  Low (below degree): 3  High (degree or equivalent): 4 | Interviews | Interpretative Phenomenological Analysis |
| Goode et al., 1996 [40]  England | The research reported here investigated how far individ­uals see themselves as exerting control over their health, and in so doing uncovered dis­crete, underlying agendas which mediate the achievement of health. | Not stated | Cross-sectional; mixed methods  Not reported | 75 participants (age range: 18 to 65 years); at least 12 female, at least 10 male  SES (occupational class):  Low (Routine and manual occupations): at least 5 participants with quotes  High (Higher managerial, administrative and professional occupations/Intermediate occupations): at least 16 participants with quotes  *Sub-sample:*  At least 2 participants aged 60+ years with quotes available; at least 2 male  Low (Routine and manual occupations): 0  High (Higher managerial, administrative and professional occupations/Intermediate occupations): 2 | Interviews | Qualitative analysis (not specified) |
| Goyder et al., 2014 [43]  England | The primary objective was to determine whether objectively measured physical activity, 6 months after a brief intervention, is increased in those receiving physical activity ‘booster’ consultations delivered in a motivational interviewing (MI) style, either face to face or by telephone. | Not stated | Cross-sectional; mixed methods  National Institute for Health Research | 26 participants (age range: 45.5 to 65.1 years); 12 female, 14 male  SES (deprived neighbourhoods according to the 2010 English IMD):  Low: 26  High: 0  *Sub-sample:*  At least 11 participants aged 60+ years with quotes available; at least 6 female, at least 5 male  SES (deprived neighbourhoods according to the 2010 English IMD):  Low: 11  High: 0 | Interviews (part of a process evaluation nested within a parallel-group randomised controlled trial) | Constant comparative method and ‘framework’ approach to analysis |
| Gray et al., 2016 [28]  Northern Ireland | This study aimed to provide a unique contribution to the litera­ture by investigating both the motives and barriers to PA engage­ment among older adults of differing SES in a developed country, Northern Ireland. | Self-determination theory  Self-efficacy theory | Cross-sectional; qualitative  Department of Education and Learning, Northern Ireland | 28 participants (low SES: mean age 72.7 ± 5.4 years; high SES: mean age 70.3 ± 4.1 years); low SES: 85.7% female; high SES: 71.4% female  SES (whether living in disadvantaged communities):  Low (members of healthy living centres): 14  High (members of the University of the Third Age): 14 | Focus groups | Thematic analysis |
| Guell et al., 2016 [36]  England | In this study, therefore, we aimed to investigate and describe in depth how active living relates to later life experiences and to aspirations and strategies of healthy ageing, using an ethnographic research design that combined interviews with participant observation. | Social theory | Cross-sectional; qualitative  Department of Health  Medical Research Council  British Heart Foundation  Cancer Research UK  Economic and Social Research Council  National Institute for Health Research  Wellcome Trust | **Qualitative component 1:**  27 participants (age range: 65 to 80 years); 12 female, 15 male  SES (occupational class):  Low (manual): 13  High (professional): 14  **Qualitative component 2:**  19/27 participants | Interviews  Participant observations | Thematic analysis |
| Guell et al., 2018 [44]  England | We aimed to develop a typology of older people’s motivations and lifelong habits of being active as a starting point to co-designing active ageing strategies in a workshop. | Not stated | Prospective; qualitative  Department of Health  Medical Research Council  British Heart Foundation  Cancer Research UK  Economic and Social Research Council  National Institute for Health Research  Wellcome Trust | **Qualitative component 1:**  27 participants (age range: 65 to 80 years); 12 female, 15 male  SES (occupational class):  Low (manual): 13  High (professional): 14  **Qualitative component 2:**  19/27 participants  **Qualitative component 3:**  13/27 participants | Interviews  Participant observations  Participant workshops | Thematic analysis |
| Hanson et al., 2016 [54]  England | The aim was to add to our understanding of non-participation in walking groups for particular social groups and thus how they can be more effectively promoted to target people in those communities who could benefit most. | Social practice perspective | Cross-sectional; qualitative  British Heart Foundation  Economic and Social Research Council  Medical Research Council  National Institute for Health Research  Wellcome Trust | 10 participants (age range: ≥ 40 years); 6 female, 4 male  SES (deprived communities, and no participants of professional/managerial backgrounds):  Low: 10  High: 0  *Sub-sample:*  5 participants aged 60+ years; 2 female, 3 male  SES (deprived communities, and no participants of professional/managerial backgrounds):  Low: 5  High: 0 | Photo elicitation interviews | Qualitative analysis (not specified) |
| Jolly et al., 2007 [34]  England | The BRUM study aimed to compare the outcomes of home- and centre-based CR in terms of cardiac risk factors and adherence to rehabilitation 6, 12 and 24 months after recruitment following MI, PTCA or CABG and to determine reasons for non-participation. | Not stated | Cross-sectional; mixed methods  National Heart Research  National Institute for Health Research | 49 participants (age range: 34 to 87 years); 16 female, 33 male  SES (deprived areas according to the IMD):  Low: 49  High: 0  *Sub-sample:*  30 participants aged > 60 years; 10 female, 20 male  SES (deprived areas according to the IMD):  Low: 30  High: 0 | Interviews (part of an individually randomised trial to explore patients’ reasons for non-participation in/non-adherence to a home- or hospital-based CR programme) | Charting |
| Lawlor et al., 2019 [52]  Northern Ireland | We developed and tested the feasibility of a PA-promoting intervention for older women within existing community groups in socio-economically disadvantaged areas. | Social Practice Theory | Cross-sectional; mixed methods  British Heart Foundation  Cancer Research UK  Economic and Social Research Council  Medical Research Council  Research and Development Division of the Public Health Agency (Northern Ireland)  Wellcome Trust | 26 participants (age range: ≥ 50 years); 26 female, 0 male  SES (areas in the most disadvantaged quartile according to the 2010 Northern Ireland Multiple Deprivation Measure):  Low: 26  High: 0  *Sub-sample:*  15 participants aged > 65 years; 15 female, 0 male  SES (areas in the most disadvantaged quartile according to the 2010 Northern Ireland Multiple Deprivation Measure):  Low: 15  High: 0 | Focus groups (part of a parallel-group delayed intervention study design to elicit participants’ views about the intervention) | Framework analysis |
| Long and Gambling, 2012 [49]  England | To examine the changes in the depth and detail of diabetes-related knowledge and confidence for persons with type 2 diabetes. | Not stated | Prospective; mixed methods  GlaxoSmith Kline  British Telecom | Interview 1: 25 participants; at least 1 female, at least 4 male  SES (deprived area):  Low: 25  High: 0  At least 2 participants aged 60+ years with quotes available; at least 2 male  SES (deprived area):  Low: 2  High: 0  Interview 2: 16 participants | Interviews (follow-up of participants recruited as part of a randomised controlled trial) | Directed content analysis |
| McDonald et al., 2015 [33]  England | This study aimed to explore and compare perceptions about how theory-based factors influence PA change during the transition from employment to retirement among individuals approaching retirement and recently retired. | Theory Domain Framework | Cross-sectional; qualitative  Biotechnology and Biological Sciences Research Council  Engineering and Physical Sciences Research Council  Economic and Social Research Council  Medical Research Council  Chief Scientist Office of the Scottish Government Health Directorates  National Institute for Health Research/The Department of Health  The Health and Social Care Research and Development of the Public Health Agency (Northern Ireland)  Wales Office of Research and Development for Health and Social Care, Welsh Assembly Government  British Heart Foundation  Cancer Research UK | 28 participants (age range: 55 to 67 years; mean age 61.00 ± 2.79 years); 15 female, 13 male  SES (2010 English IMD deciles):  Low (1-5): at least 7 participants with quotes  High (6-10): at least 15 participants with quotes  *Sub-sample:*  At least 17 participants aged 60+ years with quotes available; at least 7 female, at least 10 male  SES (2010 English IMD deciles):  Low (1-5): 6  High (6-10): 11 | Interviews | Framework analysis |
| McGowan et al., 2019 [31]  England | The present qualitative study, therefore, aims to use the TDF to elicit the views of older men and women aged 65 years and older, from diverse socioeconomic areas, concerning the acceptability of reducing their sedentary behavior. | Theoretical Domains Framework | Cross-sectional; qualitative  Not reported | 22 participants (age range: 65 to 99 years); 14 female, 8 male  SES (2015 English IMD deciles, re-categorised for consistency with other studies in systematic review):  Low (1-5): 14  High (6-10): 8 | Interviews | Thematic analysis structured using the framework approach |
| Moffatt et al., 2017 [37]  England | This qualitative study aimed to capture the experiences of patients engaged with Ways to Wellness in its first 14 months of operation and to identify the impact of the Link Worker social prescribing programme on health and well-being. | Not stated | Cross-sectional; qualitative  Cabinet Office of the UK Government  British Heart Foundation  Cancer Research UK  Economic and Social Research Council  Medical Research Council  National Institute for Health Research | 30 participants (age range: 40 to 74 years); 14 female, 16 male  SES (occupational class):  Low (Routine and manual occupations): 10  High (Higher managerial, administrative and professional occupations/Intermediate occupations): 20  *Sub-sample:*  18 participants aged 60+ years; 7 female, 11 male  SES (occupational class):  Low (Routine and manual occupations): 7  High (Higher managerial, administrative and professional occupations/Intermediate occupations): 11 | Interviews | Thematic analysis |
| Morris et al., 2022 [46]  England | This study explores how people with LTCs managed their health and wellbeing under social distancing restrictions and self-isolation during the first wave of the COVID-19 pandemic, and examines why some people were more able to manage than others. | Concept of ‘vital conjunctures’ as a lens | Cross-sectional; qualitative  National Institute for Health Research | 44 participants (age range: ≥ 40 years); 25 female, 19 male  SES (IMD deciles, re-categorised for consistency with other studies in systematic review):  Low (1-5): at least 13 participants with quotes  High (6-10): at least 3 participants with quotes  *Sub-sample:*  27 participants aged 60+ years; at least 6 female, at least 4 male  SES (IMD deciles, re-categorised for consistency with other studies in systematic review):  Low (1-5): 7  High (6-10): 3 | Interviews | Thematic analysis |
| Nimegeer et al., 2018 [45]  Scotland | The aim of the key informant interviews was to gain an overview of key environmental changes related to the new motorway. With resident interviews, the aim was to understand how participants perceived, experienced and used their neighbourhood, whether these had changed, and what role (if any) the new motorway had played in these changes. | Not stated | Cross-sectional; qualitative  Medical Research Council  National Institute for Health Research Chief Scientist Office of the Scottish Government Health Directorates  British Heart Foundation  Cancer Research UK  Economic and Social Research Council  Wellcome Trust  Scottish Government | **Qualitative component 1:**  30 participants (mean age 52 ± 15 years); 64% female, 36% male  SES (deprived neighbourhoods according to the 2012 Scottish IMD):  Low: 30  High: 0  *Sub-sample:*  At least 1 participant aged 65+ years with quotes available; at least 1 female  SES (deprived neighbourhoods according to the 2012 Scottish IMD):  Low: 1  High: 0  **Qualitative component 2:**  12/30 participants | Interviews (part of a natural experiment study)  Photo-elicitation interviews (part of a natural experiment study) | Thematic analysis |
| Ogilvie et al., 2010 [53]  Scotland | We report the qualitative findings of the baseline phase of a longitudinal mixed-method study of a new urban section of the M74 motorway in Glasgow, Scotland, that aims to combine quantitative epidemiological and spatial data with qualitative interview data from local residents. | Ecological model of behaviour | Cross-sectional; qualitative  Medical Research Council  British Heart Foundation  Department of Health  Economic and Social Research Council  Wellcome Trust  Chief Scientist Office of the Scottish Executive Health Department (now of the Scottish Government Public Health and Wellbeing Directorate) | 12 participants (age range: 34 to 72 years); 7 female, 5 male  SES (car access, and relatively deprived areas):  Low (no car access): 7  High (car access): 5  *Sub-sample:*  At least 3 participants aged 60+ years with quotes available; at least 3 female  SES (car access, and relatively deprived areas):  Low (no car access): 3  High (car access): 0 | Interviews (baseline phase of a longitudinal mixed-method study) | Thematic analysis (method of constant comparison) |
| Procter et al., 2014 [56]  England | The aim of this paper is to describe the BCTs used during the Walk to Work intervention, delivered by workplace Walk to Work promoters, and examine participants’ and promoters’ views and experiences of the different techniques. | Socio-ecological model | Cross-sectional; qualitative  National Institute for Health Research  British Heart Foundation  Cancer Research UK  Economic and Social Research Council  Medical Research Council  Welsh Government  Wellcome Trust | 22 participants, of whom 4 were promoters, 4 were promoters and intervention group participants, and 14 were intervention group participants (age range: 22 to 65 years); 10 female, 12 male  SES (annual household income):  Low (< £30,000): 8  High (≥ £30,000): 12  Note: annual household income “not given” for 2 participants.  *Sub-sample (intervention group participants):*  2 participants aged 60+ years; 0 female, 2 male  SES (annual household income):  Low (< £30,000): 0  High (≥ £30,000): 1  Note: annual household income “not given” for 1 participant. | Interviews (part of a process evaluation to explore participants and promoters’ views and experiences of the intervention) | Framework method of data management and constant  comparison |
| Rind and Jones, 2015 [35]  England | Applying focus group methodology amongst 19 participants in four groups, this study aims to unpack how broader societal and environmental changes associated with industrial decline affect beliefs and attitudes towards physical activity in ex-mining communities in the North-East of England. | Ecosocial theory  Conceptual framework linking physical activity to socio-cultural dimensions of industrial decline | Cross-sectional; qualitative  Medical Research Council  Economic and Social Research Council  Scholarship from the School of Environmental Sciences, University of East Anglia  British Heart Foundation  Department of Health  Wellcome Trust | 19 participants (mean age 72 years); 9 female, 10 male  SES (deprived region):  Low: 19  High: 0 | Focus groups | Framework of data analysis |
| Sawyer et al., 2018 [51]  Scotland | The aim of this study was to explore perceived environmental factors contributing to the creation of an activity-supportive neighbourhood in a deprived setting in the UK. | Framework of influences on physical activity and diet for ethnic minority groups | Cross-sectional; qualitative  Not reported | 23 participants (age range: 16 to 77 years); 13 female, 10 male  SES (income-deprived neighbourhoods):  Low: 23  High: 0  *Sub-sample:*  5 participants aged > 60 years; at least 2 female, at least 1 male  SES (income-deprived neighbourhoods):  Low: 5  High: 0 | Photo-elicitation interviews | Thematic analysis |
| Scott-Arthur et al., 2021 [38]  England | The aim of our study is to examine how residents of a poor urban neighbourhood understand and practice health and wellbeing, being mindful of material and symbolic constrains, alternative understandings and values and differences between groups. | Bourdieu's theory of habitus, capital and field | Cross-sectional; qualitative  Not reported | 30 participants (age range: ≥ 20 years); 15 female, 15 male  SES (deprived area according to the 2019 English IMD):  Low: 30  High: 0  *Sub-sample:*  12 participants aged 65+ years; at least 2 female, at least 4 male  SES (deprived area according to the 2019 English IMD):  Low: 12  High: 0 | Interviews (as part of an ethnographic study) | Constant comparative method |
| Tully et al., 2019 [47]  Northern Ireland | The aim of the study was to bridge the evidence gap by developing and testing the feasibility of delivering and evaluating a complex peer-led, multicomponent physical activity intervention, derived from a socioecological model of health, in socioeconomically disadvantaged community-dwelling older adults. | Socioecological model of health  Social cognitive theory | Cross-sectional; mixed methods  National Institute for Health Research  Health Improvement Division of the Public Health Agency  British Heart Foundation  Cancer Research UK  Economic and Social Research Council  Medical Research Council  Research and Development Office for the Northern Ireland Health and Social Services  Wellcome Trust | **Qualitative component 1:**  12 participants (age range: 60 to 92 years); 7 female, 5 male  SES (wards with Northern Ireland Multiple Deprivation Measure scores in the most disadvantaged quartile):  Low: 12 participants  High: 0 participants  **Qualitative component 2:**  18 participants, of whom 8 were peer mentors, 7 were intervention group participants, and 3 were control group participants (age range: 50 to 70 years); 15 female, 3 male  SES (wards with Northern Ireland Multiple Deprivation Measure scores in the most disadvantaged quartile):  Low: 18 participants  High: 0 participants  *Sub-sample (intervention group participants):*  7 participants (age range: 60 to 68 years); 6 female, 1 male  SES (wards with Northern Ireland Multiple Deprivation Measure scores in the most disadvantaged quartile):  Low: 7 participants  High: 0 participants | Interviews  Interviews and focus groups (part of a process evaluation of intervention delivery for a pilot randomised controlled trial)  Interviews only (part of a process evaluation of intervention delivery for a pilot randomised controlled trial) | Directed content analysis approach  Thematic analysis |
| Wormald et al., 2006 [48]  England | The aim of the study, therefore, was to explore participants' perceptions of the operation and effectiveness of the AL service in Kingston-upon-Hull. | Not stated | Cross-sectional; qualitative  Hull and East Riding Specialist Health Promotion Service  Eastern and West Hull Primary Care Trusts  Hull Neighbourhood Renewal Strategy | 16 participants (age range: 15 to 73 years); 11 female, 5 male  SES (deprived communities):  Low: 16  High: 0  *Sub-sample:*  At least 4 participants aged 60+ years with quotes available; at least 2 female, at least 2 male  SES (deprived communities):  Low: 4  High: 0 | Focus groups | Content analysis technique based on the 'framework' approach |
| Zandieh et al., 2016 [29]  England | This study aims to examine inequalities in perceived neighbourhood safety, pedestrian infrastructure and aesthetics in high- versus low-deprivation areas and their possible influences on disparities in older residents’ total outdoor walking levels. | Not stated | Cross-sectional; mixed methods  Erasmus Mundus scholarship supplied by the European Union | **Qualitative component 1:**  19 participants (age range: ≥ 65 years); 13 female, 6 male  SES (low- and high-deprivation areas according to the 2010 English IMD):  Low (high-deprivation areas): 10  High (low-deprivation areas): 9  **Qualitative component 2:**  52 participants (age range: ≥ 65 years); 35 female; 17 male  SES (low- and high-deprivation areas according to the 2010 English IMD):  Low (high-deprivation areas): 26  High (low-deprivation areas): 26 | Walking interviews  Questionnaire with open-ended questions | Thematic analysis |
| Zandieh et al., 2017 [30]  England | Therefore, this study aims to examine inequalities in neighborhood walkability (i.e., residential density, land-use mix and intensity, street connectivity, and retail density) in high- versus low-deprivation areas and their possible influences on disparities in older adults’ outdoor walking levels. | Not stated | Cross-sectional; mixed methods  Erasmus Mundus scholarship supplied by the European Union | 19 participants (age range: ≥ 65 years); 13 female, 6 male  SES (low- and high-deprivation areas according to the 2010 English IMD):  Low (high-deprivation areas): 10  High (low-deprivation areas): 9 | Walking interviews | Thematic analysis |

*Note:* SES, socio-economic status; UK, United Kingdom; IMD, Index of Multiple Deprivation; PA, physical activity; BRUM, Birmingham Rehabilitation Uptake Maximisation; CR, cardiac rehabilitation; MI, myocardial infarction; PTCA, percutaneous transluminal coronary angioplasty; CABG, coronary artery bypass graft; TDF, Theoretical Domains Framework; LTCs, long term conditions; BCTs, behaviour change techniques; AL, Active Lifestyles.

^a^For data collection and/or analysis.

^b^Only those relevant to the systematic review.

^c^Two participants lived in sheltered accommodation.
